# Behavioral and brain mechanisms mediating conditioned flight behavior in rats

**DOI:** 10.1038/s41598-021-87559-3

**Published:** 2021-04-15

**Authors:** Michael S. Totty, Naomi Warren, Isabella Huddleston, Karthik R. Ramanathan, Reed L. Ressler, Cecily R. Oleksiak, Stephen Maren

**Affiliations:** Department of Psychological and Brain Sciences and Institute for Neuroscience, Texas A&M University, 301 Old Main Dr., College Station, TX 77843-3474 USA

**Keywords:** Neuroscience, Learning and memory, Fear conditioning

## Abstract

Environmental contexts can inform animals of potential threats, though it is currently unknown how context biases the selection of defensive behavior. Here we investigated context-dependent flight responses with a Pavlovian serial-compound stimulus (SCS) paradigm that evokes freeze-to-flight transitions. Similar to previous work in mice, we show that male and female rats display context-dependent flight-like behavior in the SCS paradigm. Flight behavior was dependent on contextual fear insofar as it was only evoked in a shock-associated context and was reduced in the conditioning context after context extinction. Flight behavior was only expressed to white noise regardless of temporal order within the compound. Nonetheless, rats that received unpaired SCS trials did not show flight-like behavior to the SCS, indicating it is associative. Finally, we show that pharmacological inactivation of two brain regions critical to the expression of contextual fear, the central nucleus of the amygdala (CeA) and bed nucleus of the stria terminalis (BNST), attenuates both contextual fear and flight responses. All of these effects were similar in male and female rats. This work demonstrates that contextual fear can summate with cued and innate fear to drive a high fear state and transition from post-encounter to circa-strike defensive modes.

## Introduction

The selection of appropriate defensive behavior is vital to survival in the face of threat. Associative learning allows animals and humans to adapt their behavior to avoid predicted danger, and environmental contexts are critical for discriminating between fear and safety. Traumatic events can lead to pathological fear and the dysregulation of contextual processing appears to be central to various psychopathologies, such as post-traumatic stress disorder (PTSD)^1–3^. For example, context processing deficits in patients with PTSD can lead to the overgeneralization fear^4–10^, deficits in the extinction of fear^9,11–16^, and the renewal of extinguished fear in safe contexts^17^. This suggests that a complete understanding of how contexts regulate conditioned defensive behavior is essential to identifying neural circuits relevant to fear and anxiety disorders.

Pavlovian fear conditioning has been used for decades to model aversive learning and memory in rodents, and many have investigated the role of contextual cues on multiple measures of fear^1,18,19^. Previous work has revealed that contexts can both directly elicit conditioned defensive responses via contextual fear and also modulate the efficacy of another stimulus to control fear behavior without fear to the context itself, a process known as occasion setting^1,20,21^. Although conditioned fear can manifest as a wide range of behaviors, it is typically expressed as defensive freezing behavior in rodents. This raises an important question about the factors governing the selection and topography of defensive behavior under threat. An influential theory of defensive behavior, predatory imminence theory, posits that defensive behavior scales with threat proximity on a spatiotemporal scale such that freezing behavior is observed in post-encounter modes (once threat has been realized), whereas flight behavior is part of the circa-strike defensive mode (when threat is proximal)^22^. This has been demonstrated in both humans and rodents using naturalistic predator threats^23–25^, but it remains unclear whether conditioned threats, such as an auditory conditioned stimuli commonly used in Pavlovian fear conditioning, can drive circa-strike behavior such as flight. Recently, Fadok and colleagues developed a modified auditory Pavlovian fear conditioning procedure that uses a serial-compound stimulus (SCS) to elicit both freezing and flight defensive modes in mice^26^. The SCS is comprised of a pure tone stimulus immediately followed by a white noise stimulus that elicits two conditioned responses (CRs): freezing and flight behavior, respectively. Moreover, they show that the switch between freezing and flight behavior is gated by microcircuitry within the central nucleus of the amygdala (CeA), a structure critical to the expression of Pavlovian CRs^27,28^. Interestingly, SCS-evoked flight is context-dependent and only expressed in the conditioning context. This contrasts with freezing behavior, which is readily evoked by a conditioned stimulus in any context in which it is encountered. The SCS conditioning procedure thus presents a unique opportunity to investigate mechanisms by which context interacts with threat signals to select and scale defensive behavior.

In order to fully reveal the neural circuits that underly pathological fear, it is first important to understand the distinct neural circuits that underly various defensive modes and how they are gated or modulated by context^29^. Here we sought to determine the behavioral and neural mechanisms mediating the influence of context on the expression of defensive behaviors to an SCS in both male and female rats. One possibility is that the conditioning context serves as an occasion setter allowing the SCS to evoke flight behavior in the conditioning context, but not in other contexts. Another possibility is that direct context-US associations produce contextual fear that summates with that to the SCS to elevate threat imminence thereby yielding flight. The occasion setting hypothesis predicts that flight would be specific to the conditioning context and would not be expressed elsewhere, whereas the summation hypothesis predicts that flight would be evoked in any shock-associated context, regardless of whether it had hosted SCS-shock trials. In a series of experiments to test these competing hypotheses, we found that rats displayed flight behavior when the SCS was presented in a US-associated context different than the conditioning context. Moreover, extinguishing fear to the conditioning context suppressed flight behavior in that context. We further provide evidence that SCS-evoked flight is a conditioned response by showing that flight-like behavior cannot be explained by sensitization or fear-potentiated startle and is specific to white noise stimuli, which are innately aversive^30^. Finally, we show that pharmacologically inactivating either the CeA or BNST, brain regions that are critical to the expression of contextual fear, reduces flight-like behavior. All of these effects were similar in male and female rats. We thus argue that SCS-evoked flight behavior is a high fear state driven by the summation of cued, contextual, and innate fear.

## Results

### A conditioned serial-compound stimulus evokes flight behaviors in rats

**Figure 1:**
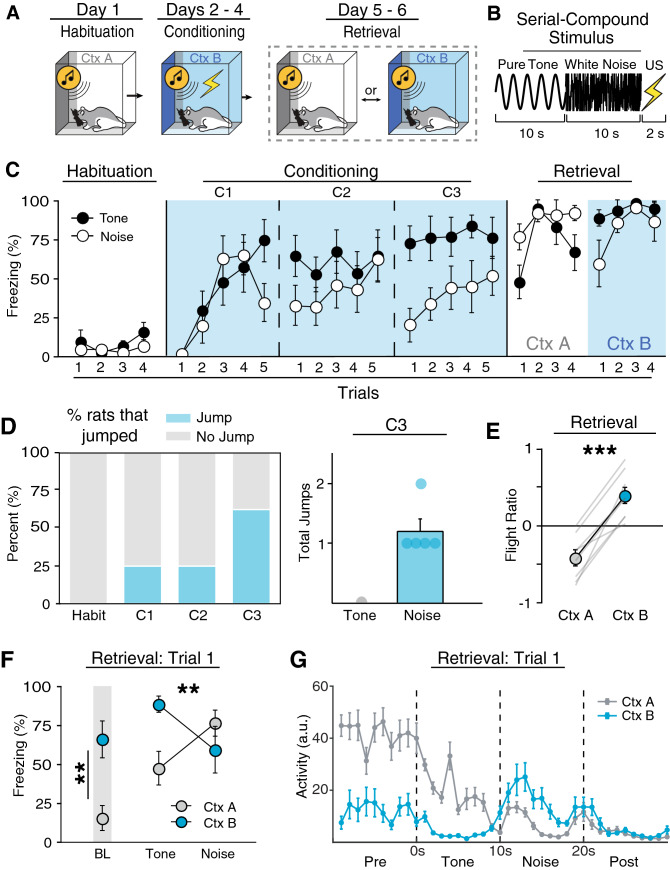
A serial compound stimulus (SCS) elicits context-dependent flight-like behavior. (**A**) Behavioral design. (**B**) Schematic representation of the SCS. (**C**) Average freezing data for tone and noise stimuli during each SCS presentation during Habituation, Conditioning, and Retrieval. Rats showed lower freezing to the noise on the second and third day of conditioning. (**D**) Percentages of rats that showed at least one jump during an SCS for each respective day of behavioral testing. Of the rats that showed at least one jump, jumps were exclusive to noise stimuli (C3). (**E**) Average flight ratio in which positive numbers represent increased movement to the noise relative to tone, whereas negative numbers represent decrease activity relative to tone. Rats displayed flight like behavior when tested in the conditioning, but not habituation context. (**F**) Average freezing data during Retrieval shows that rats tested in the conditioning context showed high freezing during baseline and the first tone presentation but decrease to the noise, whereas rats tested in the habituation context showed low freezing to baseline and the tone which increase to the noise. (**G**) Averaged motor activity data from 10 s before SCS onset to 10 s after SCS offset in both the Habituation (Ctx A) and Conditioning (Ctx B) contexts. All data are represented as mean ± SEM pooled across sex [within-subjects design (*n* = 8)]; *, **, and *** denote *p* < 0.05, *p* < 0.01, and *p* < 0.001, respectively.

Previous work shows that SCSs can evoke flight behavior in mice, but it is unknown if this behavior occurs in rats. Therefore, we first sought to determine if rats show flight-like behavior to an SCS using the behavioral protocol first described by Fadok and colleagues^26^. In this procedure (Fig. 1A), rats were first habituated to four SCS presentations (tone → white noise; each stimulus consisted of 10 s trains of 500 ms pips with an inter-pip interval of 500 ms; Fig. 1B) in context A (Day 1), then conditioned with five SCS-US presentations for the next three days in context B (Days 2–4), and finally tested with four SCS-alone presentations in both context A and B (Days 5 and 6; counterbalanced). For this experiment, we quantified (1) freezing as a percentage of time, (2) average motor activity, as well as (3) the number of jumps (all four paws leaving the floor) and darts (rapid movement from one position to another) during both tone and noise components of the SCS. Freezing and activity were quantified automatically online by digitizing voltages emitted by force transducers under each chamber; jumps and darts were scored offline from video recordings of the sessions by observer’s blind to the experimental conditions.

As shown in Fig. 1C, prior to conditioning, SCS presentations produced low levels of freezing and there was no difference in stimulus type on either freezing [*F*(1, 6) = 1.31, *p* = 0.295] or activity [*F*(1, 6) = 2.57, *p* = 0.160]. All rats showed increased freezing behavior throughout the first day of conditioning [main effect of trial, *F*(4, 24) = 8.94, *p* < 0.0001]. Although there was no main effect of stimulus type [*F*(1, 6) = 1.91, *p* = 2.16], there was a trial × stimulus type interaction [*F*(4, 24) = 3.33, *p* = 0.026] with noise producing a decrease in freezing relative to the tone stimulus on the last trial of the first conditioning session (Fig. 1C). This suggests that noise onset is associated with a reduction in freezing. Indeed, on the second day of conditioning, rats showed less freezing to the noise CS [*F*(1, 6) = 10.12, *p* = 0.019], which was mirrored by an increase in activity [*F*(1, 6) = 15.27, *p* = 0.008]. Although the rats displayed a clear switch in defensive behavior upon noise onset, the number of jumps to the noise were low with only ~ 25% of rats displaying at least one jump (Fig. 1D). During this third and final conditioning session, all rats showed an even greater decrease in freezing and increase in activity upon noise onset [Freezing: main effect of stimulus type, *F*(1, 6) = 52.23, *p* = 0.0004; Activity: main effect of stimulus type, *F*(1, 24) = 41.15, *p* = 0.001], and ~ 60% of the rats performed at least one jump. Importantly, jumps were nearly exclusive to noise presentations, with only three total jumps observed during tone presentations across all three days of conditioning. Despite a clear increase in activity, rats emitted only a small number of total jumps with only one rat jumping multiple times during the third conditioning session (Fig. 1D).

Recent reports have noted that aversive CSs can elicit darting behavior, particularly in female rats^31^. However, we seldomly observed darting behavior to the SCS in male or female rats (3 or less total darts across all animals each day). Moreover, there were no sex differences in freezing [main effect: *F*(1, 6) = 0.769, *p* = 0.414] or activity [main effect: *F*(1, 6) = 0.598, *p* = 0.469] evoked by the SCS across conditioning, consistent with previous reports that CS-elicited freezing is similar in males and females^32,33^. For this reason, data were pooled across sex when sex differences were not observed. In summary, a conditioned SCS elicits a transition from freezing to activity, which is accompanied by flight-like jumps, in both male and female rats. Nonetheless, SCS-induced jumps were infrequent and an increase in motor activity (and decrease in freezing) is the dominant mode of SCS-evoked flight-like behavior in rats, compared to frequent jumping previously observed in mice^26,34^. We therefore use white noise-evoked decreases in freezing from here on as the primary metric for flight behavior in rats.

### SCS-evoked flight behavior in rats is context-dependent

Next, we sought to determine if flight behavior in rats is context-dependent as previously shown in mice^26^. In this previous work, flight behavior was observed in the conditioning context, but not when the SCS was presented in the habituation context. To test this, conditioned rats were placed into either the habituation or conditioning context (Ctx A and B, respectively) and presented the SCS four times without the US. Although there was no difference in overall freezing between contexts [*F*(1, 6) = 0.95, *p* = 0.367] or stimulus type [*F*(1, 6) = 0.14, *p* = 0.720], rats displayed a clear decrease in freezing upon noise onset in the conditioning context (Fig. 1C,F). In contrast, they froze more to the noise presentation in the habituation context [context × stimulus type interaction, *F*(1, 6) = 17.82, *p* = 0.006]. This was again mirrored by activity levels [*F*(1, 6) = 14.12, *p* = 0.009]; rats displayed an increase in activity to noise in the conditioning context, but not the habituation context (Fig. 1G). There was no main effect of sex for either freezing [*F*(1, 6) = 0.434, *p* = 0.535] or activity levels [*F*(1, 6) = 0.770, *p* = 0.414]. Interestingly, flight only occurred during the first trial of retrieval testing and rats froze at high levels for the remainder of the test trials [main effect of trial, *F*(3, 18) = 8.25, *p* = 0.001]. This is reminiscent of the rapid extinction of flight behavior previously reported in mice^26^.

When tested in the conditioning context, rats exhibited low levels of activity to the tone but increased their activity upon white noise onset (Fig. 1G). However, in the habituation context, rats exhibited low levels of activity throughout the duration of the SCS. To further quantify this, we computed a “flight ratio”, which was the ratio of the difference of noise- and tone-evoked load-cell activity normalized by the total load-cell activity for the first retrieval test trial (further described in “Materials and methods”). This metric spans a scale from − 1 to 1 whereby increased activity during noise relative to tone yields positive values and decreased relative activity is represented by negative values. As shown in Fig. 1E, flight ratios were greater to the noise compared to the tone in the conditioning context relative to the habituation context [*F*(1, 6) = 86.26, *p* < 0.0001]. There was once again no main effect of sex [*F*(1, 6) = 0.041, *p* = 0.911]. This shows that the SCS-driven flight behavior observed in male and female rats is limited to the conditioning context. Collectively, these results demonstrate that a conditioned SCS drives flight-like behavior in rats manifest as a reduction in freezing punctuated by infrequent jumping behavior, and this pattern of responding to the SCS was context-dependent, as has previously been reported in mice.

### Flight-like behavior depends on context-US associations

We next investigated the properties of the test context that gate flight behavior. One possibility is that context serves as an occasion setter, informing the animal about the SCS-US association in the conditioning context. Alternatively, flight may be driven by a high fear state resulting from the summation of SCS-US and context-US associations. To discriminate among these possibilities, we explored whether conditioned flight would be expressed in an excitatory context that had never hosted SCS-US trials (i.e., a context in which animals experienced unsignaled shocks).

**Figure 2:**
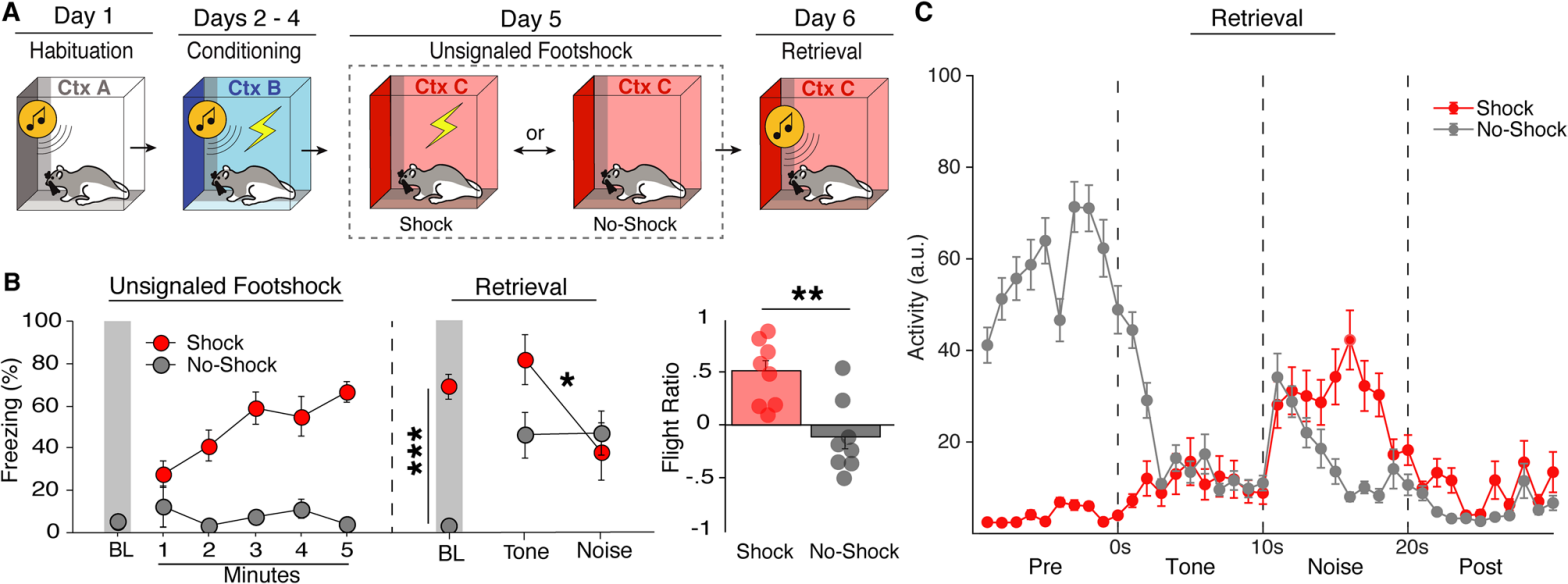
Flight-like behavior depends on context-US associations. (**A**) Behavioral design. (**B**) Average freezing data shows rats that received unsignaled footshocks (Shock) froze at high levels, whereas rats that were merely exposed to the context (No-Shock) froze at low levels. For Retrieval testing, Shock animals showed higher baseline freezing and a decrease in freezing upon white noise onset whereas No-Shock animals showed low baseline levels and remained freezing at moderate levels throughout the SCS. Average flight ratio shows that rats that Shock animals showed a bigger flight response than No-Shock animals. (**C**) Averaged activity data during the first trial of Retrieval for Shock and No-Shock animals. All data are represented as mean ± SEM pooled across sex [Shock (*n* = 8); No-Shock (*n* = 8)]; *, **, and *** denote *p* < 0.05, *p* < 0.01, and *p* < 0.001, respectively.

To this end, rats first underwent habituation and conditioning as previously described (Fig. 2A). There was once again very little freezing to the SCS prior to conditioning and no difference in stimulus type [*F*(1, 12) = 1.607, *p* = 0.229]. Rats displayed increased freezing across conditioning sessions [main effect of day: *F*(2, 24) = 9.809, *p* = 0.0008] as well as noise-elicited decreases in freezing [day × stimulus type interaction: *F*(1, 12) = 14.109, *p* < 0.0001]. Next, to test if flight depends on a context-US association, rats were separated into two groups that would either receive five unsignaled USs in a novel context (context C) or would merely be exposed to the same context for an equal amount of time (Fig. 2A, Day 5). As shown in Fig. 2B, rats that received unsignaled USs (Shock group) showed increased freezing across the session, whereas rats that were not shocked (No-Shock group) froze at low levels [minutes × group interaction: *F*(5, 60) = 11.364, *p* < 0.0001].

For retrieval testing, all rats were placed back into context C and after a 3-min baseline period were presented one SCS-alone trial. Shock animals showed much higher levels of fear to the context via freezing during the baseline period compared to No-Shock animals [*F*(1, 12) = 107.324, *p* < 0.0001]. Upon SCS presentation, Shock animals displayed a dramatic switch from freezing to activity upon noise onset (Fig. 2C), whereas No-Shock animals decreased freezing momentarily, but quickly reverted back to freezing [group × stimulus type interaction: *F*(1, 14) = 5.928, *p* = 0.0289]. This was mirrored by the flight ratio [*F*(1, 12) = 12.926, *p* = 0.0037] (Fig. 2B). In other words, conditioned animals presented the SCS in a shock-associated context displayed flight-like behavior, whereas animals tested in a neutral context did not. No sex differences were observed during retrieval [*F*(1, 12) = 0.498, *p* = 0.494] nor at any other point in this experiment. Thus, by showing that flight behavior can be evoked in a shock-associated context different from the original conditioning context, these results demonstrate SCS-driven flight-like behavior depends on context-US associations rather than occasion setting by the conditioning context. This suggests that flight to an SCS is driven by a high fear state gated by summation of SCS-US and context-US association.

### Extinguishing contextual fear reduces flight-like behavior

**Figure 3:**
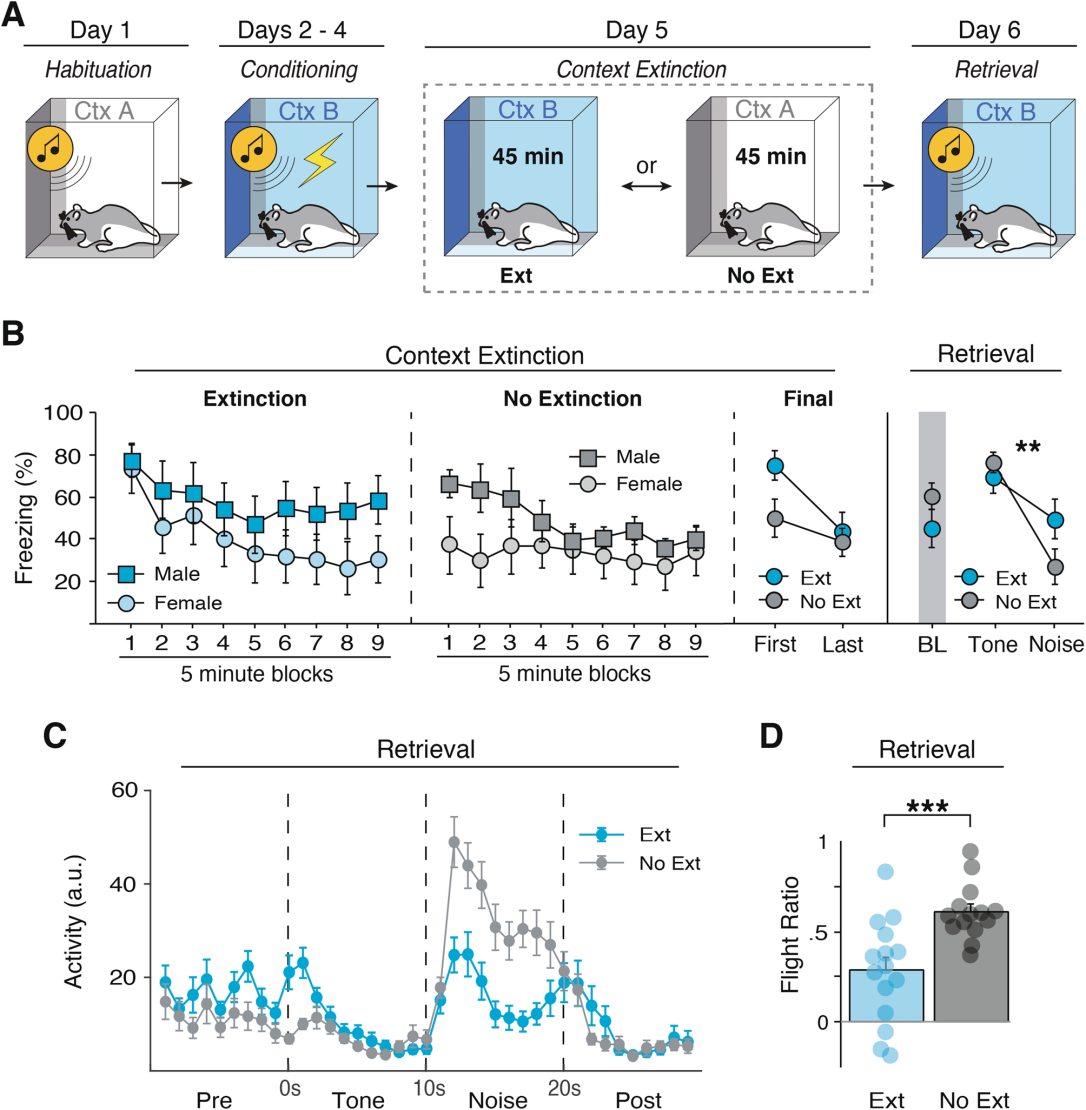
Extinguishing contextual fear reduces flight-like behavior. (**A**) Behavioral design. (**B**) Freezing data across the first extinction (Ext) or no-extinction (No-Ext) session comparing males and females averaged into 5-min blocks. Rats that underwent context extinction froze at high levels at the beginning of extinction which reduced by the end of extinction. Rats that did not undergo extinction did not show a reduction in freezing from the first to last block of context exposure. In addition, female Ext rats froze less to the conditioning context at late time points, whereas male No Ext rats showed greater generalization in the neutral context. (**C**) Ext animals did not show a significant reduction in baseline freezing, but did show a reduced flight response as shown by the reduced flight ratio. (**D**) Averaged activity data showing that Ext animals showed reduced activity during the white noise stimulus compared to No-Ext animals. All data are represented as mean ± SEM pooled across sex [EXT (*n* = 15; 7 male & 8 female); No-EXT (*n* = 14; 6 male and 8 female)]; *, **, and *** denote *p* < 0.05, *p* < 0.01, and *p* < 0.001, respectively.

If contextual fear drives flight to an SCS, then extinguishing that fear should reduce flight behavior. To test this, we habituated and conditioned rats as previously described (Fig. 3A), and then extinguished the conditioning context prior to retrieval testing. As in the previous experiments all rats showed similarly low levels of freezing to both stimuli prior to conditioning [*F*(1, 25) = 1.674, *p* = 0.2075], increased freezing across conditioning days [main effect: *F*(2, 50) = 44.685, *p* < 0.0001], and displayed noise-elicited decreases in freezing during conditioning [main effect: *F*(1, 25) = 69.535, *p* < 0.0001]. Of note, female rats in this experiment showed slightly higher levels of freezing during habituation [main effect: *F*(1, 25) = 5.208, *p* = 0.0313], but no sex differences were observed across conditioning days [*F*(1, 25) = 0.768, *p* = 0.3891].

After conditioning, rats were either placed back into the conditioning context to extinguish contextual fear for 45 min (Ext) or they were exposed to the habituation context for an equal amount of time (No-Ext) (Fig. 3A, Day 5). As shown in Fig. 3B, there was a sex difference in contextual freezing during the extinction and exposure sessions, which is consistent with previous reports^32,33^. In the conditioning context, there was a trend towards greater contextual freezing in male compared to female rats. Interestingly, this sex difference appeared late in the context extinction session, and male and female rats did not differ in the early minutes of the extinction session. There was also a sex difference in the levels of freezing among in the No-Ext groups, wherein females showed less generalization of fear to the habituation context than did males. This difference was most prominent in the earliest minutes of the No-Ext session. This differential pattern of freezing in males and females across the extinction and exposure session fell just short of significance [extinction × sex × block interaction; *F*(8, 200) = 1.926, *p* = 0.057]. Although male and female rats showed different levels of freezing during this training phase, it would not have affected subsequent SCS testing which is always conducted 3 min after placement in the test context.

A summary of the extinction data is shown in Fig. 3B. Because a subset of rats required a second extinction session, we plotted freezing during the first block of 5 extinction (or exposure) trials during the first extinction session relative to the last 5-trials (either on the first or second extinction session). Although there was not a significant group × time interaction [*F*(1, 27) = 2.994, *p* = 0.095], planned comparisons revealed that Ext animals showed a significant reduction in freezing [*F*(1, 14) = 16.930, *p* = 0.0011], whereas No-Ext animals showed stable and lower levels of freezing during the session [*F*(1, 13) = 1.611, *p* = 0.2266].

For retrieval testing, all rats were placed back into the conditioning context and presented four SCS-alone trials. Although baseline freezing was similar between groups [*F*(1, 27) = 1.857, *p* = 0.1843], NoExt rats showed a greater reduction in freezing to noise onset relative to Ext animals [stimulus type × group interaction, *F*(1, 25) = 15.880, *p* = 0.0005], which was again mirrored by changes in activity [*F*(1, 25) = 6.995, *p* = 0.0139], and flight ratio [*F*(1, 25) = 14.212, *p* = 0.0009] (Fig. 3D). In other words, context extinction reduced flight-like behavior (Fig. 3C). There was no main effect of sex for any of these metrics during retrieval testing [*F*(1, 25) = 1.388, *p* = 0.2498]. These results provide converging evidence that SCS-driven flight-like behavior is driven by summation of fear to the SCS and conditioning context.

### Flight-like responses in rats are specific to white noise and not due to sensitization

One outstanding question is whether flight behavior in rats is driven by threat imminence or stimulus salience. Predatory imminence theory posits that defensive responding scales with threat proximity, and thus, white noise may elicit flight in the SCS paradigm because it is temporally proximal to the shock US. However, recent work shows that SCS-elicited flight behavior in mice is driven by stimulus salience, specifically intensity and high frequency components of white noise^34^. Indeed, this work shows in mice that flight behavior is specific to white noise regardless of whether it precedes or follows the pure tone component of the SCS. Moreover, Hersman and colleagues show that flight behavior does not require SCS-US pairings, insofar as an unpaired SCS-US procedure also produced flight to the SCS. This suggests that sensitization or pseudoconditioning might contribute to flight to the SCS. We therefore sought to determine whether the temporal order of the stimuli in the SCS influences the emergence of flight behavior, and if flight-like behavior in rats occurs after unpaired SCS-US trials.

**Figure 4:**
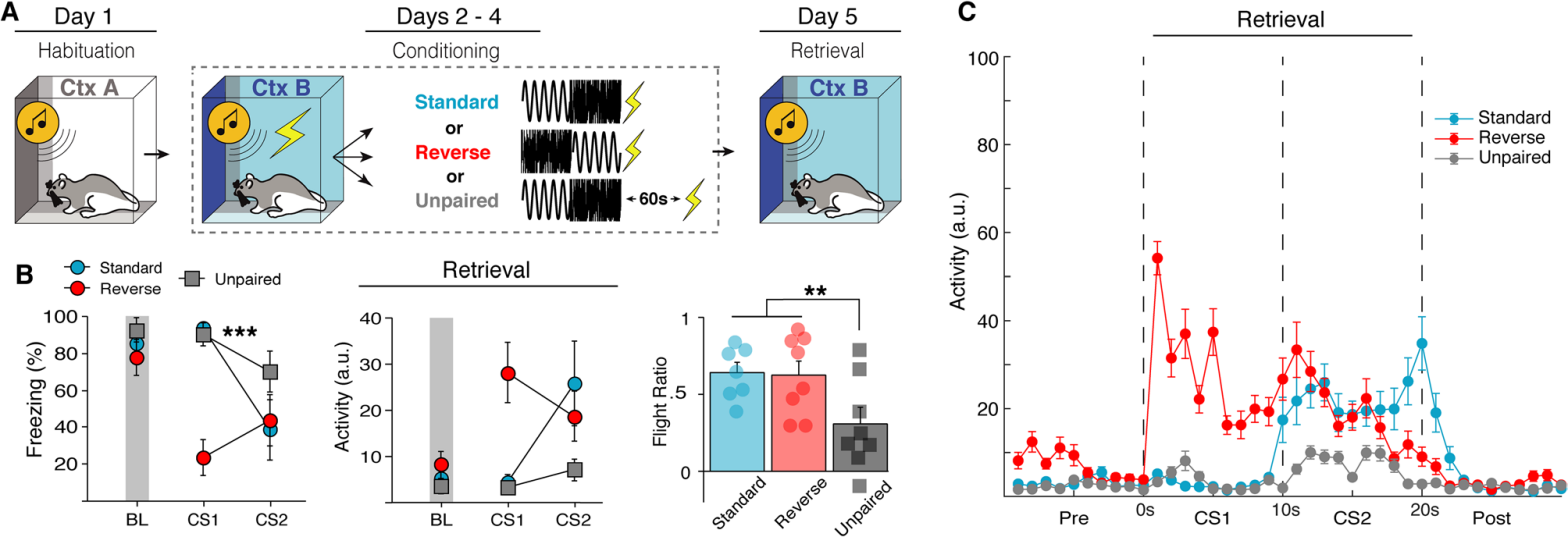
Flight-like responses in rats are specific to white noise and not due to sensitization. (**A**) Behavioral design. (**B**) Average freezing and activity data showing that flight-like behavior is specific to the white noise stimulus regardless of the temporal of order of the SCS. In a Reversed order SCS, rats show a decrease in freezing and corresponding increase in activity to the first stimuli (noise) rather than the second (tone). The data additionally show that unpairing the SCS and US with a 60-s gap (Unpaired) prevents flight-like behavior compared to Standard SCS-US controls. This all further shown by averaged activity time across time (**C**). All data are represented as mean ± SEM pooled across sex [Standard (*n* = 7); Reverse (*n* = 8); Unpaired (*n* = 8)]; *, **, and *** denote *p* < 0.05, *p* < 0.01, and *p* < 0.001, respectively.

To test this (Fig. 4A), we compared behavior to a standard SCS or a “reverse SCS”, in which the noise preceded the tone. Animals underwent habituation and conditioning with either the standard SCS-US procedure (Standard), a standard SCS with a 60-s delay before the US (Unpaired), or a reverse SCS (noise-tone) immediately followed by a US (Reversed). All groups were then tested by presenting the same SCS that they were conditioned with (either standard or reversed) without US presentation in the conditioning context. To accurately reflect freeze-to-flight transitions in this experiment the flight ratio was calculated for each group as activity during white noise relative to the 10-s period prior to white noise onset for the first retrieval test trial. Thus, for Standard and Unpaired groups the flight ratio is the same as previously described, the ratio of the difference of noise- and tone-evoked load-cell activity to the sum of noise- and tone-evoked load-cell activity; however, for the Reversed group this becomes the ratio of the difference of noise- and pre-SCS-evoked load-cell activity to the sum of noise- and pre-SCS-evoked load-cell activity.

Prior to conditioning, there was no main effect of either group [*F*(2, 17) = 0.858, *p* = 0.4416], sex [*F*(1, 17) = 2.232, *p* = 0.1535], or stimulus type [*F*(1, 17) = 0.536, *p* = 0.4742]; though female rats did show increased freezing to tone presentations across habituation which yielded a trial × sex interaction [*F*(3, 51) = 3.777, *p* = 0.0160]. All animals showed increased freezing across conditioning sessions and all groups showed decreased freezing to the noise relative to the tone, including animals in which the SCS order was reversed [day × stimulus type × group interaction: *F*(4, 34) = 15.466, *p* < 0.0001] (Fig. 4B,C). This suggests that flight responses are indeed specific to white noise, rather than determined by threat proximity. Female rats in this experiment generally showed higher freezing levels across conditioning [main effect of sex: *F*(1, 17) = 5.509, *p* = 0.0313], although no interactions with sex were observed. During retrieval testing, Unpaired animals showed a reduced flight response to white noise [main effect: *F*(2, 17) = 7.646, *p* = 0.0043] compared to both Standard [*p* = 0.0103] and Reverse groups [*p* = 0.0106] (Fig. 4B). No main effect of sex was observed [*F*(1, 17) = 1.397, *p* = 0.2535]. In summary, we show that flight behavior is exclusive to white noise in rats and that footshock sensitization cannot account for flight behavior. This reaffirms previous findings reported in mice that innately aversive auditory stimuli drive flight response in the SCS paradigm, not threat imminence.

### An unconditioned SCS fails to evoke flight behavior in a threatening context

As shown in the previous experiment, flight behavior is specific to white noise and appears to be driven by stimulus salience. This raises an alternative possibility that SCS-evoked flight may be nonassociative. White noise is commonly used as an acoustic startle stimulus and it is well known that a startle response can be potentiated when presented in a shock-associated context, a process known as fear-potentiated startle^35–37^. Although we showed in the last experiment that unpaired SCS/US presentations fail to produce robust flight behavior, an unpaired CS can come to act as a conditioned inhibitor (i.e., safety signal) which may have reduced flight behavior^38^. Thus, we next investigated whether an excitatory context might drive a potentiated startle to the noise that could account for SCS driven reductions in freezing and concomitant flight behavior.

**Figure 5:**
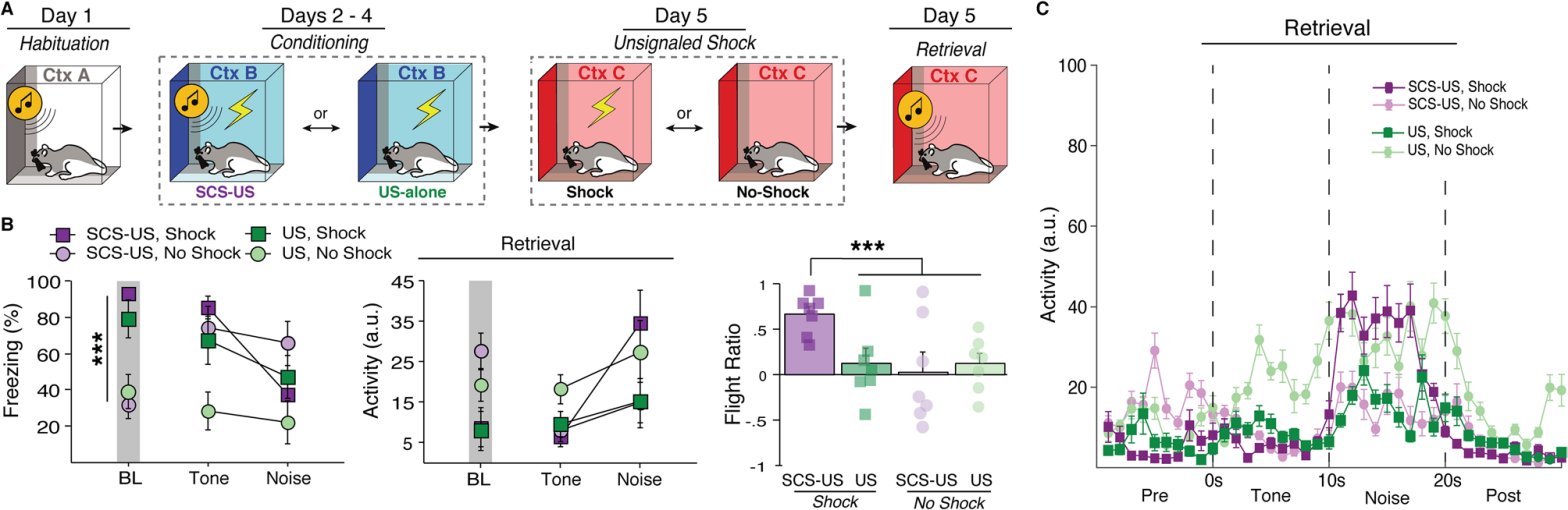
An unconditioned SCS fails to evoke flight behavior in a threatening context. (**A**) Behavioral design. (**B**) Averaged freezing and activity data showing that rats that received US-alone trials throughout conditioning (US) and tested in a US-associated context (Shock) had a reduced flight response in comparison to rats that received SCS-US pairings. This is shown by increased freezing and decreased activity to the white noise stimulus, and a reduced flight ratio. Rats that were tested in a neutral context (No-Shock) also showed reduced flight responses compared to SCS-US/Shock animals. This is further shown by the average activity trace of each group (**C**). All data are represented as mean ± SEM pooled across sex [SCS-US/Shock (*n* = 7); SCS-US/No-Shock (*n* = 7); US-alone/Shock (*n* = 7); US-alone/No-Shock (*n* = 7)]; *, **, and *** denote *p* < 0.05, *p* < 0.01, and *p* < 0.001, respectively.

All animals were first habituated with four SCS-alone trials. No differences were observed between groups [*F*(1, 24) = 0.604, *p* = 0.445], stimulus type [*F*(1, 24) = 3.205, *p* = 0.0860], or sex [*F*(1, 24) = 0.927, *p* = 0.4445]; although females showed slightly higher freezing across habituation trials [trial × sex interaction: *F*(3, 72) = 2.870, *p* = 0.0423]. To test if SCS driven flight can be explained by fear-potentiated startle (Fig. 5A), animals either underwent standard SCS-US conditioning or received an equal number of unsignaled USs across three consecutive days. The day after conditioning, animals were placed into a novel context C where they either received five US-alone presentations or context exposure, similar to Experiment 2. Finally, all animals were presented SCS-alone trials in context C on the last day of experimentation. This yields a 2 × 2 design in which animals were conditioned with either SCS-US or US-alone trials and were subsequently tested in either a threatening (Shock) or neutral context (No-Shock). If SCS driven flight is merely a potentiated startle response, we would expect both animals conditioned with SCS-US and US-alone trials to exhibit flight-like behavior in the shock-associated context.

During SCS-US conditioning, all animals showed increased freezing across sessions [*F*(2, 24) = 70.058, *p* < 0.0001] and lower levels of freezing to noise than tone [*F*(1, 12) = 29.132, *p* = 0.0002], though the difference in freezing between stimuli were similar across days [day × stimuli: *F*(2, 24) = 2.784, *p* = 0.0818]. Animals that received US-alone trials also showed increased freezing across sessions [*F*(2, 24) = 22.404, *p* < 0.0001]. No sex differences were observed during conditioning. One day after conditioning, animals either received five unsignaled footshocks in a novel context C or were exposed to the context for an equal amount of time. Animals that received footshocks showed an increase in freezing behavior relative to No-Shock animals [*F*(5, 120) = 10.762, *p* < 0.0001].

On the day of retrieval testing (Fig. 5B), Shock animals showed much higher levels of baseline freezing than No-Shock animals [*F*(1, 23) = 34.997, *p* < 0.0001] and there was no difference in SCS-US and US-alone groups [*F*(1, 23) = 0.145, *p* = 0.7070] or sex [*F*(1, 23) = 0.064, *p* = 0.8025]. At the first SCS presentation, all groups froze at a high level except for the US-alone/No-Shock group [conditioning × unsignaled shock interaction: *F*(1, 23) = 4.062, *p* = 0.0557]. Upon white noise onset, the SCS-US/Shock group showed a dramatic increase in activity greater than all other groups [flight score main effect: *F*(3, 24) = 3.632, *p* = 0.0272], including the US-alone/Shock group [*p* = 0.0193] (Fig. 5B,C). Indeed, rats in the US-alone/Shock group exhibited low levels of SCS-evoked flight that was similar to that in paired animals (SCS-US/No-Shock) that fail to express flight-like behavior in safe contexts. We therefore conclude that SCS-driven flight behavior requires an SCS-US association and cannot be attributed to sensitization or fear-potentiated startle.

### Muscimol inactivation of the central or extended amygdala attenuates flight behavior

If SCS-evoked flight depends on context-US associations, then inactivating brain regions that are critical for this process should block flight behavior. The bed nucleus of the stria terminalis (BNST) has been shown to be critical for the expression of contextual but not cued fear^39–41^, whereas the CeA is critical for both contextual and cued fear^28,42^. Based on this, we reasoned that inactivation of the either CeA or BNST would block freezing to the conditioning context and SCS-evoked flight responses.

**Figure 6:**
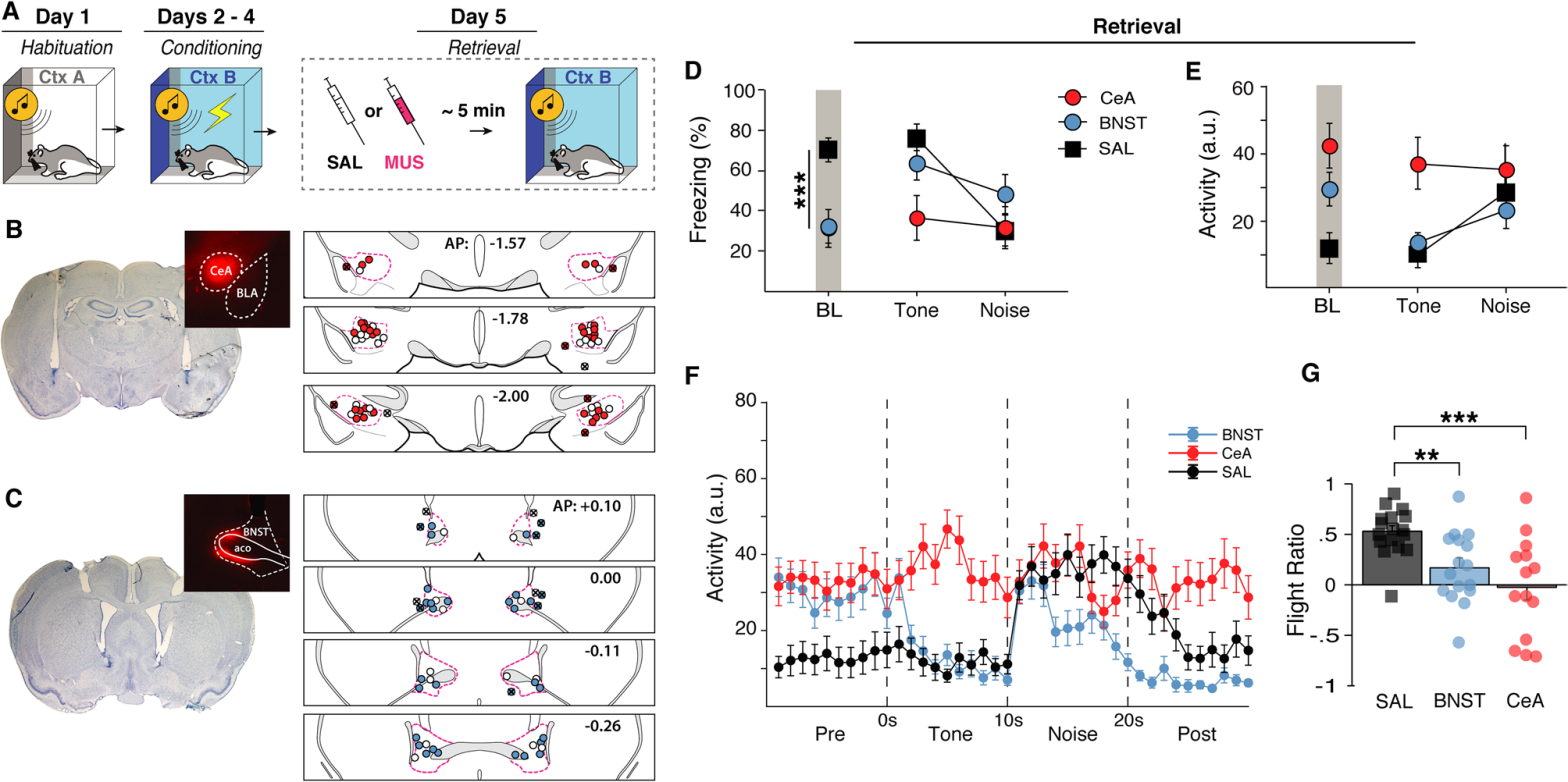
Pharmacological inactivation of either the BNST or CeA disrupts flight-like behavior. (**A**) Behavioral design. Histological summary of CeA (**B**) and BNST (**C**) cannula placements with representative thionin-stained sections and drug spread with fluorescent muscimol. Labeled anterior–posterior coordinates are relative to bregma. (**D**) averaged freezing data showing that CeA and BNST animals both showed reduced baseline freezing. BNST animals increased freezing to tone presentation and remained at higher freezing levels during white noise. CeA animals remained at low levels of freezing during the SCS. (**E**,**F**) averaged activity levels during the first SCS presentation showing that BNST animals showed a reduced flight response and CeA animals did not increase activity from tone to noise at all despite a lack of freezing behavior. This is reflected in the flight ratio (**G**). All data are represented as mean ± SEM pooled across sex [CeA (*n* = 14); BNST (*n* = 16); SAL (*n* = 16)]; *, **, and *** denote *p* < 0.05, *p* < 0.01, and *p* < 0.001, respectively.

All rats were implanted with cannula targeting either the BNST or CeA one week prior to SCS habituation and conditioning (Fig. 6A–C). During habituation, there were main effects of stimulus type [*F*(1, 40) = 5.145, *p* = 0.029] and trial [*F*(3, 120) = 3.284, *p* = 0.023] with animals showing increased freezing to tones at the end of habituation. All animals showed increased freezing to the SCS across conditioning days [*F*(2, 80) = 117.994, *p* < 0.0001] with decreased freezing to the white noise stimulus [main effect of stimulus type: *F*(1, 40) = 356.902, *p* < 0.0001]. No sex differences were observed across habituation or conditioning.

Immediately prior to retrieval testing rats received local infusions of either the GABA_A_ agonist muscimol (MUS) or saline (SAL). Control rats with cannula targeting either the BNST or CeA were pooled to create a single SAL group. Inactivation of either the CeA [*p* = 0.0013] or BNST [*p* = 0.0012] reduced baseline freezing relative to SAL controls [*F*(1, 40) = 0.901, *p* = 0.3482], an indication of diminished contextual fear. During subsequent presentations of the SCS, CeA animals showed lower freezing to the tone [main effect: *F*(2 40) = 5.457, *p* = 0.008] compared to both SAL [*p* = 0.0028] and BNST animals [*p* = 0.038] (Fig. 6D). Upon noise onset, there was not a stimulus type × group interaction [*F*(1, 40) = 0.901, *p* = 0.3482], but planned comparisons reveal that SAL animals [*p* < 0.0001], but not CeA [*p* = 0.6165] or BNST [*p* = 0.1321], showed a reduction in freezing behavior (Fig. 6D). This was reflected in the flight ratios [main effect: *F*(2, 40) = 8.773, *p* = 0.0007], which showed that both CeA [*p* < 0.0001] and BNST inactivation [*p* = 0.0067] blunted flight behavior (Fig. 6G). This was also evident in averaged activity plots (Fig. 6E,F). There were no sex differences in these effects. In summary, inactivating either the CeA or BNST was sufficient to block both contextual fear and SCS-evoked flight responses. This provides further evidence that SCS-evoked flight depends on neural circuits that regulate contextual fear.

## Discussion

Here we investigated context-dependent flight behavior evoked by a serial-compound stimulus conditioning procedure in male and female rats. First, we show that context-dependent flight-like behavior can be evoked in rats using the SCS procedure, although jumping was less frequent than previously reported in mice^43^. We further found that flight occurs in any shock-associated context and that extinguishing contextual fear to the conditioning chamber suppresses flight-like behavior, demonstrating that SCS-evoked flight behavior reflects the summation of cued and contextual fear. Although flight is specific to white noise, we found that fear-potentiated startle and sensitization could not account for SCS-evoked flight. That is, neither unpaired nor neutral SCS presentations were sufficient to drive flight behavior in a shock-associated context, even when animals had received an equal number of prior footshocks. Finally, we show that pharmacological inactivation of brain regions that are critical for the expression of contextual fear, either the CeA or BNST, is sufficient to block the expression of flight behavior. Together, these data demonstrate that conditioned flight behavior in the SCS paradigm is driven by a high fear state via a combination of contextual and cued fear.

Until now only mice had been used to study flight responses in the SCS paradigm and it was unknown if rats would also elicit flight behavior to an SCS^26,34,44^. We found that the SCS procedure indeed evoked context-dependent flight responses similar to reports in mice, although jumping is less frequent in rats. One important limitation of our study is that we used indirect measures of motion, opposed to other work that used direct measurements of speed via camera which makes direct comparisons of locomotion difficult^26,44^. Despite this, visual comparison of the increase in activity observed here to those in previous reports appear similar^26,34,44^. Additionally, increases in activity and jumping observed here were both specific to the white noise stimulus. It is currently unclear why rats display infrequent jumps although we speculate that this may be a species-specific difference. Because rats in these experiments were single-housed, it is not known whether paired housing would affect SCS-evoked flight behavior. Environmental enrichment has been shown to increase context conditioning and discrimination^45,46^, but also reduces overall conditioned fear^46^. Investigating additional parameters that might increase jumping and other escape-like responses to a SCS in rats would be worthwhile for studying conditioned circa-strike behavior in rats.

Previous work suggests that female rats display more active defensive behaviors, such as defensive darting, in response to an aversive CS^31^. Based on this, we expected that female rats may be more likely to show flight-like behavior compared to males in the SCS paradigm. However, we did not observe significant sex differences in SCS-evoked jumping or activity levels. Darting behavior was seldom observed during SCS presentations in both female and male rats. This could be due to scoring differences as original reports used an automated detection method^31^. Conversely, other work shows that female mice actually exhibit increased freezing in the SCS paradigm, though the average speed of male and female mice during the SCS did not differ^47^. We also report here that female rats showed increased conditioned freezing under some conditions. Specifically, females exhibited elevated freezing to the white noise-tone (‘reverse SCS’) compound during both habituation (no-shock) and conditioning. This suggests that this stimulus may have been more aversive to female rats compared to males, although no sex differences in freezing or flight were observed to the reversed SCS during retrieval testing.

Consistent with earlier reports^32,33^, we also observed a sex difference in contextual freezing during the context extinction manipulation illustrated in Fig. 3. Female rats showed reduced freezing late in the extinction session, and also exhibited reduced generalization of context fear from the conditioning context to the habituation context. It is unlikely that these sex differences in contextual freezing affected behavior to the SCS, because SCS trials were delivered shortly (3 min) after animals were placed in the test context (a period of time during which sex differences in contextual freezing were not apparent). Ultimately, understanding sex differences in fear memory and behavior is critical to developing therapeutics for PTSD and other trauma-related disorders that may have sex-linked genetic predispositions^48,49^.

As others have shown (Hersman et al., 2020), we found that rats only display flight-like behavior to white noise, even when it was the first element of the SCS compound. This supports previous work demonstrating that stimulus salience determines flight behavior in mice^34^. This previous work specifically shows that it is the high-frequency component and intensity (> 80 dBs) of white noise that evokes flight. Indeed, loud, high-frequency stimuli appear to innately produce flight behavior in mice. However, it was also shown that sensitization by previous US presentations actually reduces the frequency of flight behavior due to increased competition with freezing behavior^50^.

In the present work, we show that flight is not due to sensitization or fear-potentiated startle. So, how do SCS-US associations, innately aversive white noise stimuli, and fearful contexts interact to drive flight-like behavior in rats? We believe that the most parsimonious explanation is that SCS-evoked flight results from the summation of cued, contextual, and innate fear; although all three are not always necessary to elicit flight behavior. For example, flight in mice can be evoked innately to loud, high-frequency stimuli (i.e., without conditioned fear)^50^. Additionally, flight to an SCS can be evoked without a salient high-frequency component by increasing the intensity above 90 dBs^34^. In the SCS paradigm, auditory stimuli are presented at 75–80 dB which appears to be just below the threshold to innately evoke flight responses to white noise^26,34^. Coupled with our findings that SCS flight requires both SCS-US and context-US associations, we propose that cued and contextual fear sum with salient stimuli to cause freeze-to-flight transitions.

In line with the behavioral results, we found that reversible inactivation of either the CeA or BNST was sufficient to disrupt both contextual freezing and context-dependent flight responses. The finding that CeA inactivation disrupts both contextual and cued fear is supported by decades of work demonstrating that the CeA is critical to the expression of conditioned responses^27,28,51^. Moreover, the role for the CeA in flight-like behavior in rats is consistent with earlier work in mice^26^. This work shows that SCS-evoked flight is gated by corticotrophin-releasing hormone (CRH+) neurons in the CeA that inhibit somatostatin-expressing neurons that are critical for freezing behavior^52–55^. Similarly, our finding that inactivating the BNST disrupts defensive freezing to the conditioning context, but not the SCS, is in line with previous work showing that the BNST affects freezing to unpredictable threats^39–41,56–60^.

Considering this, what is the neural mechanism for cross-modal summation of fear that gates freeze-flight transitions to an SCS in a shock-associated context? One possibility is that BNST projections to the CeA drive CeA CRH+ neurons to gate flight behavior^26,61–63^. By this view, summation of contextual and SCS-evoked fear occurs within the CeA, and at some threshold leads to a suppression of freezing and a transition to flight. Alternatively, flight may be regulated by projections from both the BNST and CeA that converge in the periaqueductal grey (PAG), a midbrain structure that is critical to defensive responding^64–66^. For example, stimulation of the dorsal PAG can result in both flight and freezing behavior, whereas stimulation of the ventral PAG drives freezing^67–71^. Indeed, recent work in mice has shown that the dorsal PAG performs a synaptic threshold mechanism for computing escape behavior^72^. Based on this, the PAG may integrate convergent CeA and BNST inputs to summate aversive context and SCS-evoked fear to drive flight behavior^73^. Future work should investigate these pathways and their potential role in mediating flight behavior in the SCS paradigm.

To summarize, we have shown that rats display flight-like behavior to an SCS, although rats show less frequent escape-like behaviors, such as jumping and darting, compared to mice. Flight-like behavior evoked by the SCS is specific to white noise, gated by contextual fear, and cannot be accounted for by sensitization or fear-potentiated startle. We conclude that SCS conditioning results in a high fear state driven by the summation of cued, contextual, and innate fear that drives a freeze-to-flight transition. Although freezing behavior is a robust post-encounter response that scales with threat imminence^22^, the present data reveal that an SCS can suppress freezing under a high fear state in a context-dependent manner. Specifically, animals exhibit freezing to an SCS in a familiar, safe context, but will transition to flight-like behavior in a shock-associated context. This transition occurs in contexts in which the SCS has never been paired with shock, suggesting that it results from a summation of conditioned fear to the SCS and excitatory context. Under these conditions, the SCS may model aspects of predator contact that evoke circa-strike behavior. Future work should investigate the neural mechanisms underlying the transition from post-encounter to circa-strike defensive behaviors and how this is driven by the summation of fear to conditioned and innately aversive stimuli^22,29^. This may reveal important clinical implications for psychiatric disorders that are characterized by high fear states and the dysregulation of contextual processing, such as panic disorder and PTSD^1,74^.

## Materials and methods

### Subjects

Experiments used adult Long-Evans rats (*n* = 163) acquired from Envigo (Indianapolis, IN; 200–240 g upon arrival). Males and females were used in equal numbers throughout all experiments. All animals were housed in a climate-controlled vivarium and kept on a fixed light/dark cycle (lights on starting at 7:00 a.m. and off at 9:00 p.m.; experiments took place during the light phase of the cycle). Rats were individually housed in clear plastic cages (with bedding consisting of wood shavings; changed weekly) on a rotating cage rack. Group assignments for behavioral testing was randomized for cage position on the racks. Animals had access to standard rodent chow and water ad libitum. Animals were handled by the experimenter(s) (~ 30 s/day) for five consecutive days prior to the start of any surgeries or behavior. All procedures were in accordance with the US National Institutes of Health (NIH) Guide for the Care and Use of Laboratory Animals, ARRIVE guidelines (https://arriveguidelines.org), and were approved by the Texas A&M University Institutional Animal Care and Use Committee.

### Apparatuses

All behavioral testing occurred within one of two rooms in the laboratory. Each behavioral room housed eight identical rodent conditioning chambers (30 cm × 24 cm × 21 cm; MED Associates, Inc.). Each chamber was housed in a larger, external sound-attenuating cabinet. Rear walls, ceilings, and the front doors of the testing chambers were made of Plexiglas, while their sidewalls were made of aluminum. Grid floors of the chambers were comprised of 19 stainless steel bars (4 mm in diameter) and spaced 1.5 cm apart (center to center). The grid floors were attached to an electric shock source and a solid-state grid scrambler for delivery of the US (MED Associates, Inc.). A speaker attached to each chamber was used to deliver the auditory CS. As needed for each context, the chambers were equipped with 15 W house lights, and small fans were embedded in the cabinets (providing background noise of ~ 70 dB). An aluminum pan was inserted beneath the grid floor to collect animal waste. A small camera was attached to the top of the cabinet for video monitoring of behavior.

Measurements of freezing and motor activity were performed using an automated system^75^. Specifically, each behavioral testing chamber rested on a load-cell platform that was sensitive to cage displacement due to each animal’s movements. During behavioral testing, load-cell activity values (ranging from − 10 to + 10 V) were collected and digitized at 5 Hz using Threshold Activity Software (MED Associates, Inc.). Offline conversions of the load-cell activity values were performed to generate absolute values ranging from 0 to 100; lower values indicate minimal cage displacement, which coincided with freezing behaviors in the chambers. Accordingly, freezing bouts were defined as absolute values of ≤ 10 for 1 s or more. The percentage of freezing behavior during the pre-SCS baseline and SCS trials was computed for each behavioral session. Motor activity was analyzed by directly reporting the absolute values generated by the Threshold Activity Software (i.e., larger values indicated more movement in the cage). Jumping and darting behavior were manually scored off-line from video recordings by an experimenter blind to experimental conditionings.

Unique contexts (A, B, and C) were used for various phases of behavior testing. Chamber assignments were unique to each context and group assignments were counterbalanced across test chambers when possible. For each experiment contexts A and B were assigned to different behavioral testing rooms. For context A, the test chamber and pans beneath the grid floors were wiped down with an ammonium hydroxide solution (1%). The cage lights were turned off, chamber fans were turned on, and the cabinet doors were left open. Black Plexiglas panels were also placed over the grid floors. The behavioral room was lit with white light (red lights were turned off). Animals were transported to and from the chambers using white plastic transport boxes. For context B, an acetic acid solution (3%) was used to wipe down and scent the chambers, the cage lights were turned on, the chamber fans were turned off, and the cabinet doors were closed. The behavioral room was lit with dim red light (white room lights were turned off). Rats were transported to and from context B using black plastic transport boxes that included a layer of clean bedding. For context C, an ethanol solution (70%) was used to wipe down and scent the chambers, the cage lights were turned on, the chamber fans were turned on, and the cabinet doors were open. The behavioral room was lit with white lights (red lights remained off) and rats were transported to and from context C in white plastic transport boxes with clean bedding. Testing in context C was always performed in the same behavioral room as context A.

### Experimental design

Overviews of each behavioral experiment are provided in the figures [behavioral schematics were composed in Adobe Illustrator CC 23.0.1 (http://www.adobe.com/products/illustrator) and all illustrative content is original work by the authors]. The auditory serial-compound stimulus (SCS) used for all experiments was comprised of ten 500 ms pure tone pips (80 dB, 7 kHz) presented at a frequency of 1 Hz (500 ms inter-pip intervals, 10 s total length) and immediately followed by ten 500 ms white noise pips (80 dB, 1–20 kHz) presented at a frequency of 1 Hz (500 ms inter-pip intervals, 10-s total length). During conditioning the SCS was paired with a mild unconditioned footshock stimulus (US, 1.0 mA, 2 s). Unsignaled footshocks used for Experiments 2 and 4 were of the same duration and intensity. Intertrial-intervals (ITI) were always 60 s.

### Surgery

Rats were anesthetized with isoflurane (5% induction, ~ 2% maintenance), the top of their heads were shaven, and they were placed in a stereotaxic mount (Kopf Instruments, Tujunga, CA). A small incision was made with a scalpel, fascia lining the skull was scrubbed away with cotton swabs, and the scalp was retracted with forceps. The skull was leveled horizontally before burr holes were drilled above either the BNST or CeA. Four additional holes were made anteriorly and posteriorly (two each) for skull screws. After skull screws were placed, two stainless-steel cannulas (26 gauge, 8 mm; Plastics One) were lowered into either the CeA (target coordinates; ML: 4.0, AP: − 2.0, DV: − 8.0) or the BNST (target coordinates; ML: 1.5, AP: 0.0, DV: − 6.5). Cannula targeting the BNST were inserted at a 10° angle to avoid rupturing the ventricle. Thus, angled coordinates used during stereotaxic surgery targeting the BNST were as follows: ML: 3.13, AP: 0.0, DV: − 6.19 (ML: medial–lateral, AP: anterior–posterior, DV: dorsal–ventral). All coordinates are in reference to the skull surface at bregma. Cannula were then affixed to the skull with dental acrylic and a stainless-steel dummy (30 gauge, 9 mm; Plastics One) was inserted into the guide cannula. Rats were allowed to recover for ~ 1 week after surgery before behavioral testing.

### Drug microinfusions

The day of retrieval testing in Experiment 6, rats were placed into 5-gallon white buckets and moved into a room adjacent to the vivarium for microinfusions. Dummy cannula internals were removed and a stainless-steel injector (33 gauge, 9 mm; Plastics One) connected to polyethylene tubing was inserted into the guide cannula. Polyethylene tubing was connected to 10-μl Hamilton syringes that were mounted in an infusion pump (Kd Scientific). Muscimol was diluted to a concentration of 0.1 μg/μl in sterile saline. Infusions were made a rate of 0.3 μl/min for 1 min and the injectors were left in place for 2 min post-infusion to allow for adequate diffusion. Each infusion was verified by movement of an air bubble that separated the drug or sterile saline from distilled water within the polyethylene tubing. Clean dummy internals were inserted into each guide cannula after infusions. All infusions were made ~ 5 min prior to behavioral testing.

### Histology

Twenty-four hours after retrieval testing animals were sacrificed to confirm cannula placement. Animals were overdosed with sodium pentobarbital (Fatal Plus, 100 mg/ml, 0.7 ml), transcardially perfused with ice-cold saline and fixed with 10% physiological formalin. Perfused brains were placed in physiological formalin for 14–24 h before being moved to a 30% sucrose solution for a minimum of three days. After three days, or until brains had sunk in 30% sucrose, all brains were frozen and sectioned at − 20° on a cryostat at a thickness of 40 μm. Sections were mounted onto gelatin subbed slides, thionin stained (0.25%) to better visualize cannula placement, cover-slipped with Permount (Fisher Scientific), and then imaged on a wide-field stereoscope.

A subset of animals infused with fluorescent muscimol to verify drug spread. These animals were overdosed with sodium pentobarbital and infused with 0.3 μl of fluorescent muscimol (BODIPY TMR-X conjugate; Thermo Fisher Scientific) at a rate of 0.3 μl/min. A rest period of 2 min was given post-infusion and then animals were immediately sacrificed. Non-perfused brains were placed in a physiological formalin solution for 14–24 h before being placed in a 30% sucrose-formalin solution for a minimum of three days. These brains were also sectioned at − 20° on a cryostat at a thickness of 40 μm. Sections were then mounted onto subbed slides, coverslipped with fluoromount (Diagnostic Bio-systems), and imaged on a fluorescent microscope at 10× resolution. Hits were confirmed by verifying that the tip of the infusion needles was within the CeA or BNST. Only animals that had bilaterally confirmed placements were included in statistical analyses. Thus, animals in which the tip of either one or both cannulas were outside of the CeA or BNST were excluded from analyses.

### Exclusions

Animals were excluded if they were either statistical outliers (> 2 standard deviations from the group mean) or had missed cannula placements. For the context extinction experiment (Fig. 3), two animals (*n* = 2) were excluded from the Ext group and one animal (*n* = 1) from the No-Ext group for being statistical outliers, resulting in the following group sample sizes: Ext (*n* = 14) and No-Ext (*n* = 15). For the experiment using the reversed order SCS (Fig. 4), one animal (*n* = 1) was excluded from the Standard group for being a statistical outlier, resulting in the following group sizes: Standard (*n* = 7), Unpaired (*n* = 8), and Reverse (*n* = 8). For the fear-potentiated startle experiment (Fig. 5), one animal (*n* = 1) from each group was removed for being a statistical outlier, resulting in equal group sizes (*n* = 7). For the muscimol experiment (Fig. 6), four animals (*n* = 4) were excluded from the BNST group, four from the CeA group (*n* = 4), and 8 from the SAL group (*n* = 8) for missed cannula placements, resulting in the following group sizes: BNST (*n* = 16), CeA (*n* = 14), and SAL (*n* = 16).

### Statistical analyses

All freezing and raw threshold data were analyzed offline by custom written Python and MATLAB scripts before eventual statistical testing in StatView software. If no sex differences were observed, data were collapsed across sex in order to increase statistical power. All data were submitted to repeated or factorial analysis of variance (ANOVA) as described for each experiment. Fisher’s protected least significant difference (PLSD) test was used for post hoc comparisons of group means following a significant omnibus *F* ratio in the ANOVA (α was set at 0.05). No statistical methods were used to predetermine group sizes (group sizes were selected based on prior work and what is common for the field). Sex was included as a biological variable for all statistical comparisons. Data distributions were assumed to be normal, but these were not formally tested. All data are represented as means ± SEM.
